# Tetramethylpyrazine Alleviates Endothelial Glycocalyx Degradation and Promotes Glycocalyx Restoration via TLR4/NF-κB/HPSE1 Signaling Pathway During Inflammation

**DOI:** 10.3389/fphar.2021.791841

**Published:** 2022-01-03

**Authors:** Jin Lei, Peng Xiang, Shengmei Zeng, Le Chen, Lei Zhang, Zhiyi Yuan, Jun Zhang, Tingting Wang, Ruihong Yu, Wanping Zhang, Issa Issoufou Ibrahim, Limei Ma, Chao Yu

**Affiliations:** ^1^ College of Pharmacy, Chongqing Medical University, Chongqing, China; ^2^ Chongqing Key Laboratory for Pharmaceutical Metabolism Research, Chongqing, China; ^3^ Institute of Life Sciences, College of Pharmacy, Chongqing Medical University, Chongqing, China

**Keywords:** endothelial glycocalyx, TMP, HPSE1, TLR4/NF-κB signaling pathway, LPS

## Abstract

Tetramethylpyrazine (TMP), a Chinese traditional herbal extraction widely used in treating cardiovascular diseases, could attenuate vascular endothelial injuries, but the underlying mechanism remains incomprehensive. Vascular glycocalyx coating on the endothelium would be damaged and caused endothelial dysfunction in the inflammatory microenvironment, which was the initial factor of morbidity of many vascular diseases, such as atherosclerosis (AS). Here, we thoroughly investigated the molecular mechanism of TMP on vascular endothelial glycocalyx in the LPS-induced inflammatory model both *in vitro* and *in vivo*. Results showed that pretreatment with TMP significantly inhibited glycocalyx degradation and monocytes adhesion to the endothelial process. Moreover, TMP pretreatment inhibited the expression of HPSE1 (a major degrading enzyme of endothelial glycocalyx), Toll-like receptor 4 (TLR4), and the translocation of nuclear factor kappa B p65 (NF-κB p65). We were utilized withTLR4 siRNA, NF-κB inhibitor, and HPSE1 overexpression analysis confirmed TMP’s protection on endothelial glycocalyx injury, which further contributed to the monocyte-endothelial adhesion process. It was indicated that TMP might suppress glycocalyx degradation through TLR4/NF-κB/HPSE1 signaling pathway. Taken together, our results enriched the occurrence molecular mechanism of glycocalyx shedding and molecular regulation mechanism of TMP in protecting integrity of the glycocalyx structure during inflammation. As TMP is currently used in clinical applications, it may be considered a novel strategy against atherosclerosis through its ability to protect endothelial glycocalyx.

## Introduction

Cardiovascular disease (CVD) is the leading cause of death globally, accounting for one-third of deaths each year (Benjamin et al., 2019; Mensah et al., 2021). Alterations in the endothelial monolayer, especially endothelial cells (EC) dysfunction, is the crucial and initial steps for the vascular injury (Nieuwdorp et al., 2005; Fels and Kusche-Vihrog, 2019). The endothelium lines the vascular interior and regulates vascular stiffness for healthy cardiovascular function. Its proper function relies on the glycocalyx coating, which mediates endothelial cells (ECs) signaling and remodeling and exhibits protective and regulatory functions in vascular health (Cheng et al., 2016). In CVD procession, EC dysfunction always coincides with shedding of the EC glycocalyx coat (Hopkins et al., 2018; Kiyan et al., 2019; Yamaoka-Tojo, 2020). Additionally, a compromised glycocalyx has been shown to increase the permeability of inflammatory cells infiltrating under endothelium. Therefore, a potential avenue for treating CVD is to target ECs as well as their glycocalyx, rather than simply utilizing the systemic and medicinal treatments that are common today (Smith et al., 2011; Cheng et al., 2016).

EC-glycocalyx is mainly comprised of a network of core proteins and branching glycosaminoglycan polymers such as heparan sulfate (HS), chondroitin sulfate (CS), and hyaluronans (HA), respectively (van den Berg et al., 2003; Lipowsky, 2012; Yamaoka-Tojo, 2020; Urschel et al., 2021; Wijesekara et al., 2021). The most common of which is HS, which are anchored to endothelial cells (ECs) via transmembrane proteoglycans and always released from the endothelium upon glycocalyx injury. Therefore, level of HS is always useful as an endothelial glycocalyx injury marker. Heparanase 1 (HPSE1), the only known endo-β-d-glucuronidase capable of degrading heparan sulfate chains in mammals, was upregulated in many inflammatory diseases including cancer, diabetes, and atherosclerosis (Kiyan et al., 2019). Studies have found that inhibited heparanase activation could protect pulmonary endothelial glycocalyx integrity and reduce endothelial dysfunction (Wang et al., 2016; Huang et al., 2018), which indicated that inhibition of HPSE1 activity or upstream regulatory mechanisms might be helpful in inhibiting the vascular disease progression.

It is well-established that the signaling cascade initiated by the innate immune arm involving Toll-like receptor 4 (TLR4) is activated during inflammatory responses and is considered to be closely related to atherosclerosis (Manago et al., 2019). Knockdown of TLR4 gene expression by siRNA attenuated HG-induced inflammation, leukocyte adhesion, as well as glycocalyx dysfunction (Pahwa et al., 2016). Furthermore, TLR4-mediated response of EC to LPS was related to the activation of HPSE1. Inhibition of HPSE1 reduced LPS-mediated TLR4 activation and protected endothelial glycocalyx injury (Kiyan et al., 2019). Besides TLR4, the downstream NF-κB signaling was also capable of releasing pro-inflammatory cytokines and accelerated aortic atherosclerotic lesions process (Michelsen et al., 2004; Leng et al., 2019).

Tetramethylpyrazine (TMP), a major active ingredient extracted from *Ligusticum chuanxiong Hort*, is an alkaloid monomer and widely used in the clinical treatment to protect against coronary heart disease, diabetes, cancer, and liver injury (Zhao et al., 2016; Duan et al., 2020). Research has demonstrated TMP exerts various pharmacological effects such as anti-inflammatory (Zhang et al., 2020), anti-angiogenesis, and antioxidant (Yuan et al., 2018), which are important determinants in the outcome of glycocalyx injury. However, the effect of TMP on the endothelial glycocalyx has not been extensively studied. The aim of our present study was therefore to investigate the effects and mechanisms of action of TMP on endothelial glycocalyx.

Here, we evaluated the effect of TMP on the endothelial glycocalyx and its possible related mechanisms *in vivo* and *in vitro*. We anticipate that this work will provide a new theoretical basis for drug development that targets the endothelial glycocalyx, and it will also extend our knowledge of TMP to treat CVD and other diseases involving endothelial glycocalyx damage.

## Materials and Methods

### Chemical and Reagents

TMP (purity: >98%) was purchased from Aladdin (China). Lipopolysaccharide (LPS) and Alcian blue 8GX and Acridine orange were obtained from Sigma (United States). Foetal Bovine Serum (FBS) was purchased from Biological Industries (Israel). Albumin Borine V (BSA) was purchased from Solarbio (China). Calcein AM was purchased from Beyotime. Trizol was purchased from Invitrogen (United States).

### Cell Culture and Experimental Groups

Human umbilical vein endothelial cells (HUVECs) were obtained from ScienCell Research Laboratories, Inc. (United States) and cultured in ECM medium supplemented with 5% fetal bovine serum, 1% growth factors, and 1% antibodies (penicillin/streptomycin) in a 37°C, 5% CO_2_ incubator. THP-1, human monocyte cell line, were purchased from BeNa Culture Collection (China) and cultured in RPMI 1640 medium supplemented with 10% fetal calf serum, 1% antibiotics (penicillin/streptomycin) in a 37°C, 5% CO_2_ incubator. EA. hy 926 cells were from BeNa Culture Collection (China) and cultured in DMEM medium supplemented with 10% fetal bovine serum in a 37°C, 5% CO_2_ incubator. Based on published studies of dose methods (Huang et al., 2018), or the LPS stimulation group alone, endothelial cells were stimulated with LPS at the concentration of 0, 0.2, 0.5, 1, 2, 5 μg/ml for 12 h. For TMP pretreatment, the HUVECs were separated into four groups: control group, LPS group, and LPS&TMP (50, 200 μM) groups. The control group was cultured with a complete culture medium without LPS treatment. For the LPS group, HUVECs were treated with LPS (1 μg/ml) for 12 h. In the LPS&TMP groups, HUVECs were pretreated with TMP (50, 200 μM) for 12 h prior to LPS (1 μg/ml) stimulation for 12 h.

### Cell Viability Assay

For cell viability assays, HUVEC cells were seeded in 96-well plates at 1 × 10^5^/ml using Cell Count Kit 8 (CCK-8) and incubated overnight at 37°C. Then the cells were subjected to LPS treatment (0–5 μg/ml) for 12 h, TMP treatment (0–500 μM) for 12 h, or TMP (50,200 μM) pretreatment for 12 h and then co-treatment with LPS (1 μg/ml) for 12 h, followed by the addition of CCK-8 reagent to the cell culture medium (Huang et al., 2018). After incubation for 3 h, absorbance values were measured at 450 nm and cell viability was calculated.

### Animals and Experimental Groups

Six-week-old male C57BL/6 mice (15–20 g) were purchased from Chongqing Medical University Animal Center. The mice were housed in a specific sterile facility under controlled conditions (12 h light-dark cycle; 25±2°C). Based on published studies of dose methods (Qing et al., 2019), our experiments were performed using the following subgroups: For TMP pretreatment, mice were randomly separated into four groups (n = 5): the control group, LPS group, and LPS&TMP (3, 6 mg/kg) groups. The LPS&TMP groups received intraperitoneal injection of TMP for 7 days, and the control group and LPS group received the same amount of sterile saline instead. Then, mice in the LPS group and LPS&TMP (3, 6 mg/kg) groups were intraperitoneally injected with LPS (2.5 mg/kg) to induce a vascular injury model. After infusion of LPS for 12 h, mice were anesthetized with 4% chloral hydrate and sacrificed. Blood samples and aortic tissues were collected for further experiments. All animal experiments were undertaken with review and approval from the Animal Ethical and Experimental Committee of Chongqing Medical University.

### Western Blotting

After 12 h of TMP pretreatment and subsequently12 h with LPS treatment, cells were lysed using cell lysis buffer, and protein concentrations were measured with a BCA assay kit (Beyotime, China). The aorta tissues were collected and homogenized with RIPA buffer. Equal amounts of denatured protein were subjected to 10% SDS-PAGE and blotted onto PVDF membranes (Millipore, United States). Antibodies against β-Actin (Santa Cruz Biotech, United States), MD-2 Monoclonal Antibody (Invitrogen, United States), and Toll-like Receptor four and Phospho-NF-κB p65 (Beyotime, China) were used as primary antibodies. HPSE1 (Signalway Antibody, United States), VCAM-1 (Servicebio, China), Syndecan-1 (Abcam, United States), HS (Amsbio, United Kingdom) and horseradish peroxidase-conjugated goat Anti-rabbit IgG and horseradish peroxidase-labeled Goat Anti-Mouse IgG (Beyotime, China) were used as the secondary antibody. All the primary antibodies were diluted to 1:1000 and the secondary antibodies were diluted to 1:8000. Detection was performed using an ECL kit (Biosharp, China), according to manufacturer’s instructions. The relative amount of protein was determined by densitometry using ImageJ software.

### qPCR Assay

Total RNA from cells was extracted by TRIzol method. The RNA was reverse transcribed by Evo M-MLV Mix Kit with gDNA Clean for qPCR (Accurate Biotechnol, China). Real-time PCR was performed with SYBR^®^ Green Realtime Master qPCR Mix (TsingKe Biotech, China). The levels of ACTIN mRNA were used as endogenous control. The primers used were shown in Supplementary Table S1. The relative mRNA expression values were calculated in accordance with 2^−∆∆Ct^ methods.

### Immunofluorescence Assay

HUVECs were seeded on the coverslips in 24-well plates and cultured overnight at 37°C. Then the cells were divided into control group, LPS group, TMP group, and TMP&LPS groups and were treated as described in method 2.2. Further the cells were fixed with 4% paraformaldehyde for 15 min and then permeabilized with 0.1% Triton X-100 and blocked with 5% BSA for 1 h. The primary antibody was prepared in 1% BSA: WGA Lectin FITC (Gene Tex, United States). Incubation of the primary antibody was done at 4°C for one night. The secondary antibody was also prepared in 1% BSA: FITC-goat anti-mouse IgG, and Dylight 680-goat anti-rabbit (Proteintech, United States), the specimens were rinsed with PBS for three times and then incubated with corresponding fluorescent secondary antibody at room temperate for 1 h. Finally, the specimens were counterstained with DAPI for 8 min at room temperate. Images were captured by using a fluorescence microscope (Leica, Japan). Fluorescence images were analyzed using ImageJ software.

### Monocyte Adhesion Assay

The cells were cultured according to the grouping method in 2.2, after a series of treatments, THP-1 cells were labeled with Calcein AM (Beyotime, China) and incubated on top of a monolayer of HUVECs for 1 h. Nonadherent cells were washed 3 times with phosphate-buffered saline. Monocyte adhesion was photographed using an Olympus inverted fluorescence microscopy.

### Transfection

SiRNA and plasmid (TsingKe Biotech, China), DNA transfection reagent (Genomtech, China), and Lipofectamine 3000 (Invitrogen, United States) were used to transfect plasmid. The procedure was carried out according to the instructions. The sequences of the siRNAs used were as follows: TLR4-Homo (F: 5′-GAA​GUU​GAA​CGA​AUG​GAA​UTT-3’; R: 5′-AUU​CCA​UUC​GUU​CAA​CUU​CTT-3′).

### Electron Microscopic Visualization of the Endothelial Glycocalyx

According to the previous literature, the intravascular glycocalyx observed by transmission electron microscopy (TEM) can be labeled by the combination of Alcian blue 8GX and acridine orange staining (Zhang et al., 2019). Briefly, the mice were anesthetized and fixed to a surgical plate, exposing the heart and aorta, perfused from the left ventricular tip, and the right atrium was incised for fluid outflow. HEPES buffered saline containing 0.1% BSA (pH 7.4) was perfused at a flow rate of 8 ml/min for 15 min. Then, the perfusate was changed to phosphate buffer (pH 7.4) containing 5 mM MgCl_2_, 0.01% acridine orange, and 0.05% Alcian blue 8GX for 30 min at room temperature. After completion of that perfusion, the aorta was harvested and fixed in 2.5% glutaraldehyde in 0.1 M PBS buffer (pH 7.2) and 1% osmium tetroxide, and subjected to a series of dehydration. To prepare samples for TEM imaging, each specimen was embedded in epoxy resin. Ultrathin sections (90 nm), stained with uranyl acetate and lead citrate, were then examined by using TEM imaging (Japan Electronics JEM1200ex).

### Detection of Biochemical Parameters

Blood samples were collected from orbit and centrifuged at 7500 rpm for 15 min at 4°C for various biochemical assays, such as Aspartate Transaminase (AST), Alanine aminotransferase (ALT), Albumin (ALB), Blood Urea Nitrogen (BUN), and Creatinine (CREA).

### Statistical Analysis

Data denote mean ± S.E.M. from experiments of at least three different HUVEC donors or five independent C57BL/6 mice donors. Statistical comparisons between two independent groups were performed with Student’s t-test while one-way ANOVA tests were used to evaluate statistical differences between two conditions. *p* values <0.05 were considered statistically significant.

## Results

### LPS Induced Glycocalyx Degradation and Increased Monocyte Adhesion to HUVEC in a Dose- and Time-Dependent Manner

**FIGURE 1:**
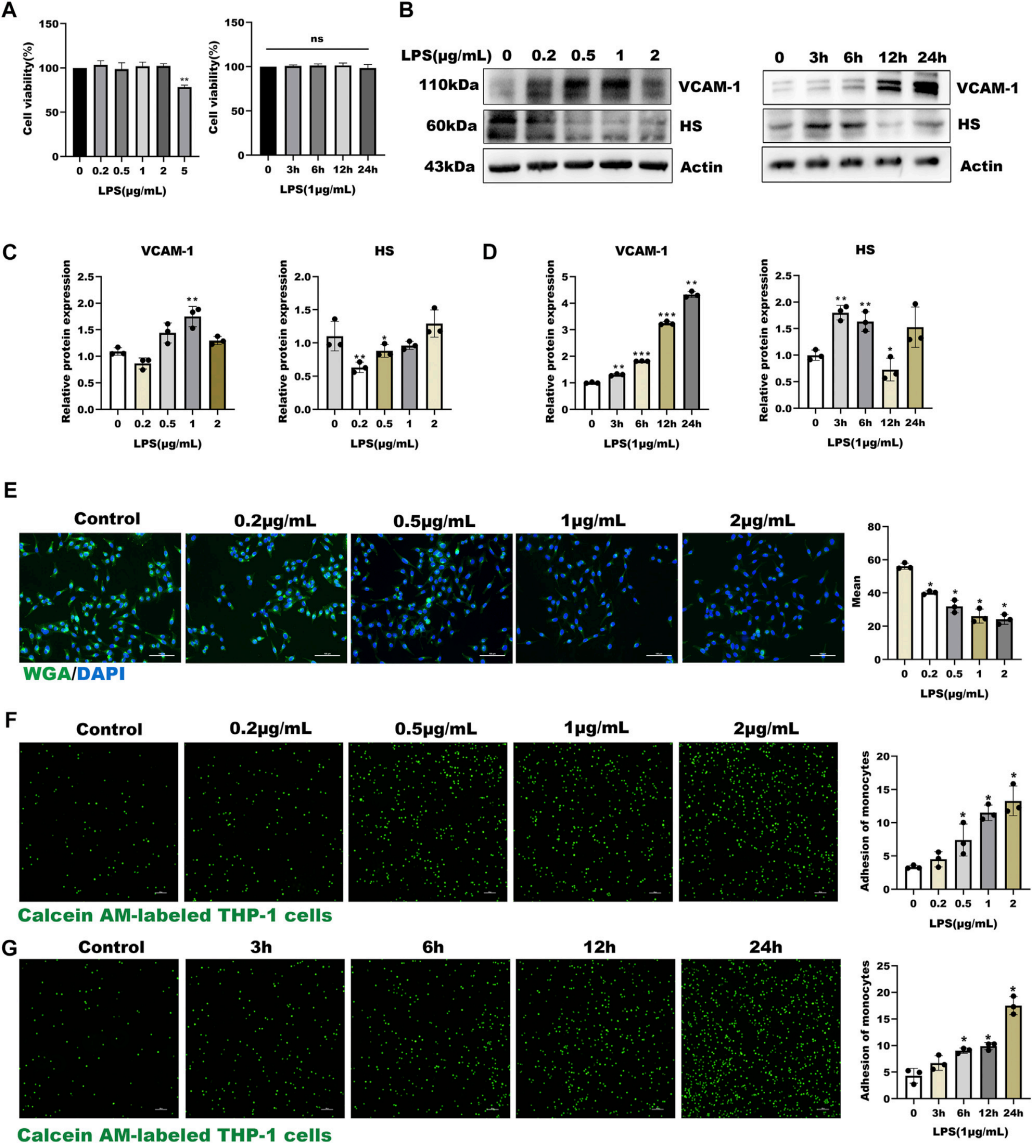
LPS induced glycocalyx loss and endothelial dysfunction in HUVEC **(A)** Effects of HUVEC treated with LPS at different concentrations and time on cell viability **(B–D)** Western blot analysis of VCAM-1 and HS in HUVEC treated with LPS at different concentrations and time **(E)** HUVEC total glycocalyx level were also detected by fluorescence microscopy analysis using WGA Lectin FITC (Green), DAPI (Blue), and Mean fluorescence quantitative statistic (*n* = 3) **(F–G)** HUVEC were treated with LPS at different concentration and time. Monocyte adhesion to endothelial cells was quantified *via* monocyte adhesion assay. Calcein AM-labeled THP-1 cells were incubated with HUVEC. Bar: 100 μm *p < 0.05. **p < 0.005; ***p < 0.001.

In the present study, HUVECs were first stained with Von Willebrand Factor (VWF), a marker of endothelial properties, and further used LPS as an inflammatory stimulator to develop a model of inflammatory-induced inflammation glycocalyx injury. Before studying the effect of LPS on glycocalyx degradation, we first investigated the cytotoxicity of LPS on HUVEC. As shown in Supplementary Figure S1A and Figure 1A, HUVECs had positive endothelial property, and LPS significantly decreased the cell viability compared with the control group (*p* < 0.01) in a time and dose-dependent manner. As shown in Figures 1B–D, the expression of HS was down-regulated in a dose- and time-dependent manner after LPS treatment, and the expression of inflammatory factor VCAM-1 was up-regulated. Further, the WGA staining assay indicated that LPS stimulated glycocalyx shedding of HUVECs (Figure 1E). Monocyte adhesion experiments showed that monocyte adhesion increased with LPS concentration, and the adhesion increased in a time-dependent manner under fixed LPS concentration (Figures 1F,G). Collectively, these results suggest that LPS promoted glycocalyx degradation and increased monocyte-endothelial cell adhesion in a time- and dose-dependent manner.

### TMP Pretreatment Protected the Glycocalyx Degradation in LPS-Induced Inflammatory Model

**FIGURE 2:**
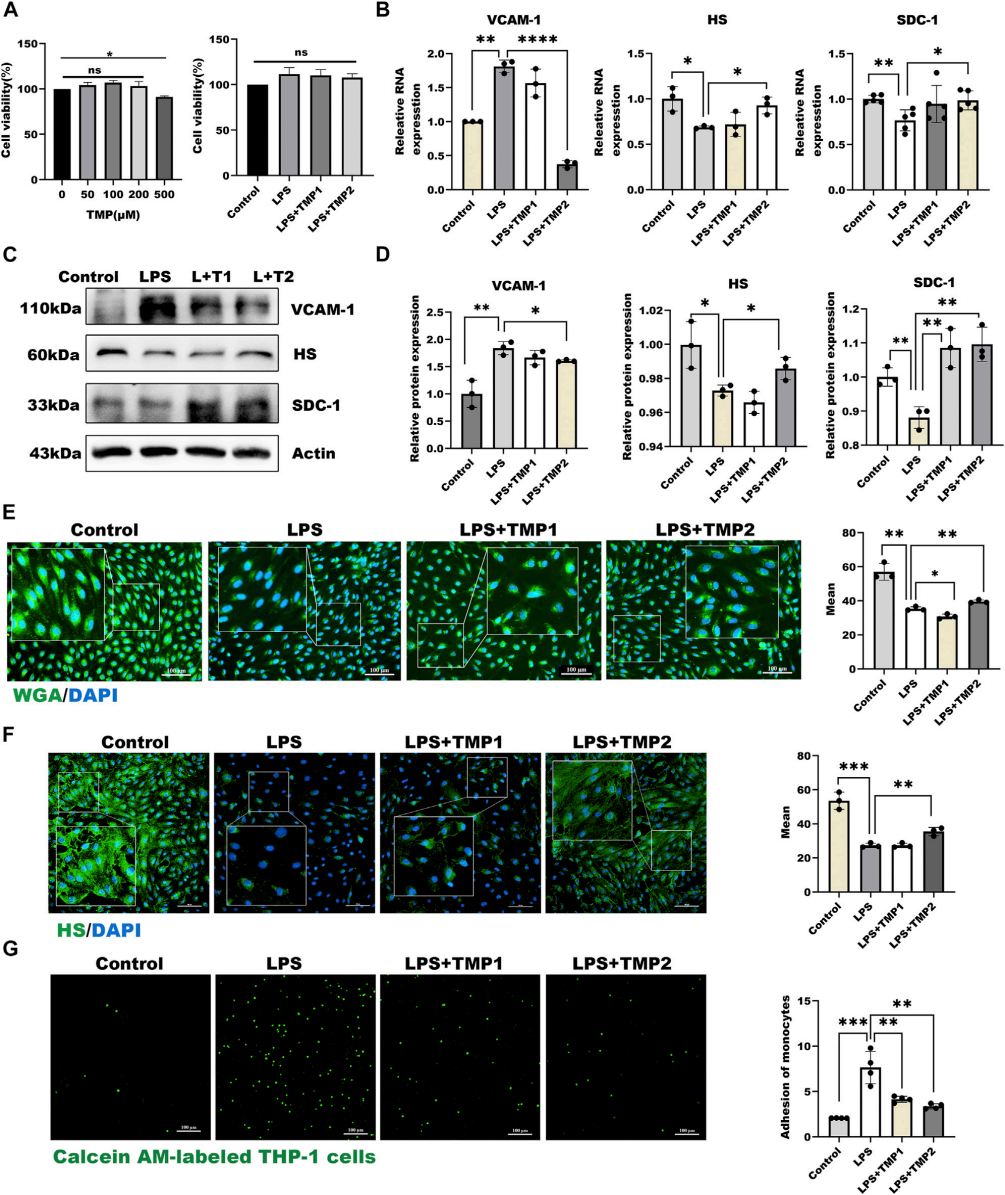
TMP pretreatment protected glycocalyx degradation in LPS-induced inflammatory model **(A)** On the left are effects of HUVEC treated with TMP at different concentrations on cell viability. On the right are effects of TMP-LPS co-treatment of HUVEC on cell viability **(B)** Real-time PCR for analysis of the glycocalyx major components HS and SDC-1, inflammatory factor VCAM-1 mRNA levels after LPS and TMP stimulation in HUVEC **(C)** Western blot analysis of the glycocalyx major components HS and SDC-1, inflammatory factor VCAM-1 in HUVEC treated with LPS and TMP **(D)** Actin was used as an internal reference, and the gray value of protein bands was quantified by ImageJ. Data are shown as mean ± SD (n = 3) **(E)** HUVEC total glycocalyx level were also detected by fluorescence microscopy analysis using WGA Lectin FITC (Green), DAPI (Blue) and Mean fluorescence quantitative statistic (*n* = 3) **(F)** HUVEC major glycocalyx component HS were detected by fluorescence microscopy analysis using HS (Green), DAPI (Blue) and Mean fluorescence quantitative statistic (*n* = 3) **(G)** HUVEC were treated with LPS at different concentration and time. Monocyte adhesion to endothelial cells was quantified via monocyte adhesion assay. Calcein AM-labeled THP-1 cells were incubated with HUVEC. Bar: 100 μm *p < 0.05. **p < 0.005; ***p < 0.001.

To study the effect of TMP on endothelial glycocalyx, we firstly investigated the cytotoxicity of TMP to HUVECs. As shown in Figure 2A, TMP did not display any cellular toxicity against the HUVECs at the concentrations of 0–200 μM, while cell viability treating with TMP in 500 μM was significantly decreased by 12% compared with the control group. Further LPS combined with TMP with 50 and 200 μM also have no detected inhibition on cell viability. As shown in Figures 2B–D, pretreatment with TMP (50, 200 μM) may remarkably inhibit endothelial glycocalyx damage in the LPS-induced glycocalyx degradation model. Both mRNA and protein levels of HS, as well as SDC-1, increased by 55%, 51% respectively in the group pretreated with TMP, relative to that in the LPS group. In addition, the mRNA and protein levels of VCAM-1 also decreased by 80%, 25% compared with the LPS group. Furthermore, the morphological observation of glycocalyx detected by immunofluorescence showed that endothelial glycocalyx was obviously abscised after LPS stimulation compared with the control group. Pretreatment with TMP, glycocalyx degradation, and HS abscission were significantly decreased by 25% and 37% (Figures 2E,F). Also, TMP exerted potent inhibition on the monocyte-endothelial adhesion process (Figure 2G). As VE-cadherin has been reported to be involved in this process, we also evaluated the effect of TMP on expression of VE-cadherin. As shown in Supplementary Figures 1B,C, TMP exerted promotion on VE-cadherin levels strongly. The preceding results indicated that pretreatment with TMP could protect the glycocalyx damage and further improve endothelial function.

### Pretreatment of TMP Exerts Its Effects by Inhibiting Glycocalyx Degrading Enzyme HPSE1

**FIGURE 3:**
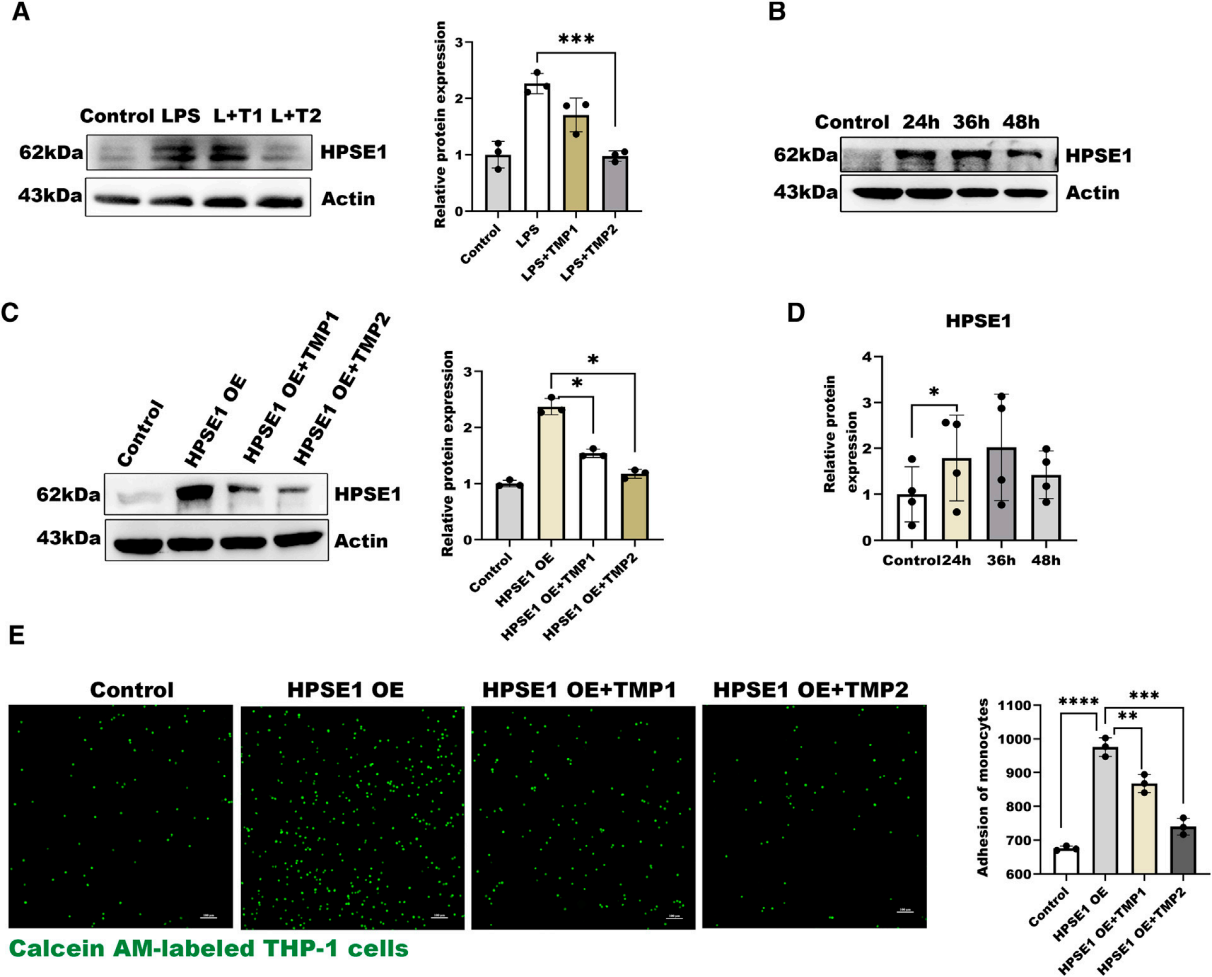
Effect of TMP on HPSE1 activation **(A)** Western blot analysis of HPSE1 expression after TMP pretreatment and LPS treatment and quantitative analysis **(B)** Western blot was used to analyze the transfection efficiency of HPSE1 at 24, 36 and 48 h after transfection **(C)** Pretreatment of HUVEC with TMP for 12 h inhibited the expression of HPSE1 24 h **(D)** The protein bands of **(B)** were quantitatively analyzed by ImageJ **(G)** Monocyte adhesion assay was used to detect the adhesion of endothelial cells 24 h after overexpression of HPSE1. Bar: 100 μm *p < 0.05. **p < 0.005; ***p < 0.001.

In the present study, we also found the expressions of HPSE1 by LPS stimulation were significantly inhibited with increasing concentration of TMP for pretreatment (Figure 3A), which indicated that TMP may reduce HPSE1 activation and repair endothelial glycocalyx degradation. Furthermore, we constructed an HPSE1 over-expression plasmid to confirm our predictions. The results in Figures 3B,D showed an increase of HPSE1 expression after transfection for 24 h accompanied. The results showed after 12 h TMP pretreatment also inhibits HPSE1 overexpression (Figure 3C) and with an increasing ratio in endothelial adhesion to monocytes, which was subsequently inhabited by TMP pretreatment 12 h (Figure 3E). These results verified that TMP down-regulated HPSE1 expression and protected endothelial function upon inflammatory activation.

### TMP Pretreatment Exerted Protection on Endothelial Glycocalyx Degradation Through TLR4/NF-κB/HPSE1 Signaling Pathway

**FIGURE 4:**
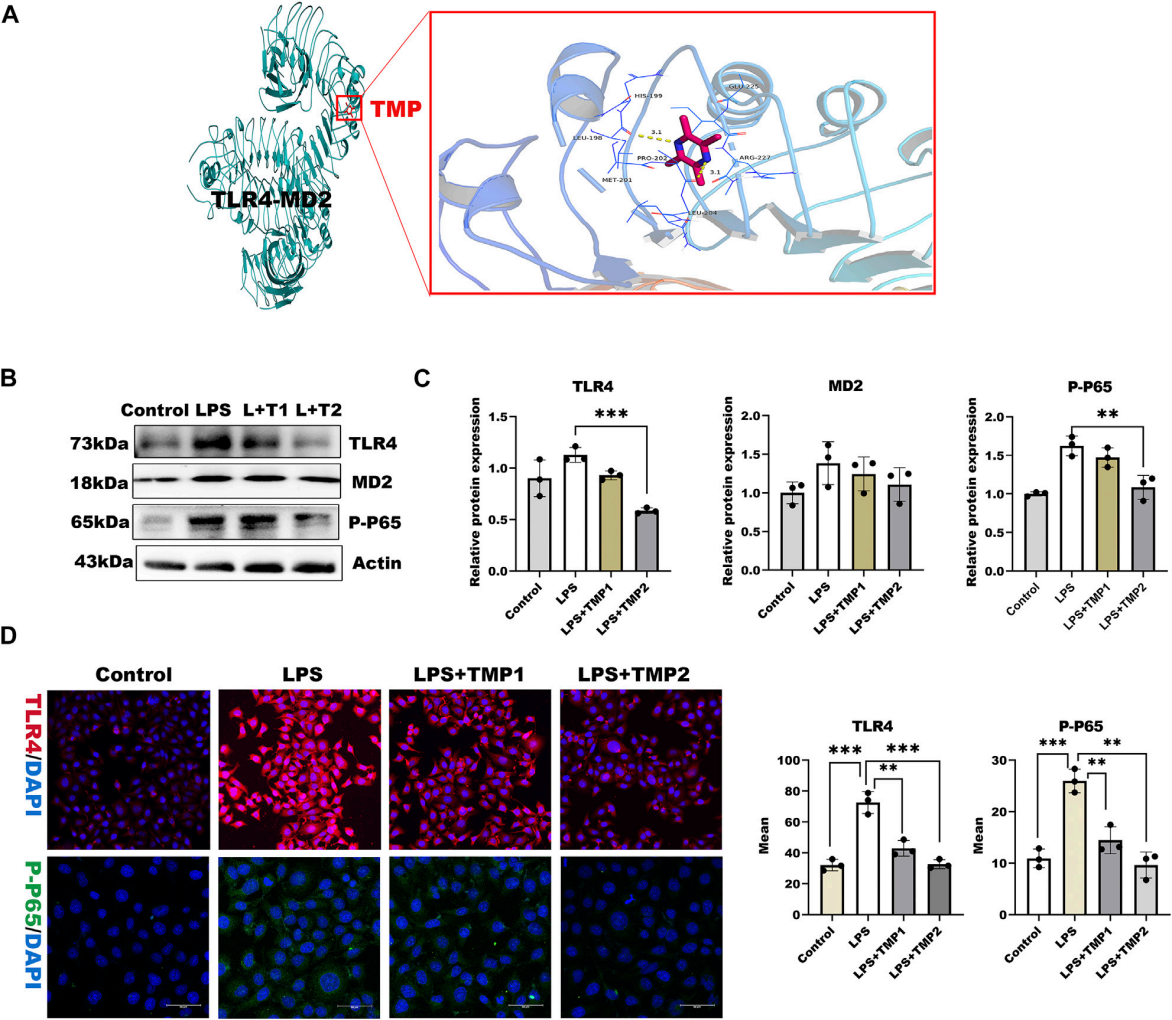
TMP exerts protection on glycocalyx through TLR4/NF-κB pathway **(A)** TMP was docked with TLR4-MD2 complexes by molecular docking technique **(B)** Western blot analysis of Proteins on the signaling axis in HUVEC treated with LPS and TMP **(C)** Quantitative analysis of protein bands were performed by ImageJ software (D) HUVEC were detected by fluorescence microscopy analysis using TLR4 (Red), phosphorylated NF-κB (Green) and DAPI (Blue) and Mean fluorescence quantitative statistic (*n* = 3). Bar: 100 μm *p < 0.05. **p < 0.005; ***p < 0.001.

To investigate the mechanisms by which TMP affects HPSE1 activation and protect against glycocalyx injury, we firstly applied molecular docking technology using PyMOL software for scoring its binding site as well as complementary values at the binding sites. As shown in Figure 4A, TMP docked into the cavity of protein TLR4/MD2 complex with a reasonable fit. The binding energy predicted by Autodock is −3.41 kcal/mol for TMP-TLR4/MD2 (Supplementary Table S2). To confirm if TMP directly targets TLR4/MD2 for further signaling activation, western blotting and immunofluorescence analysis were applied to detect levels of TLR4/MD2 in HUVECs. As shown in Figures 4B–D, the protein expression of TLR4, MD2 as well as its fluorescence intensity were both decreased in presence of TMP treatment compared with LPS group. Our results also verified P65-NF-κB was decreased upon TMP pretreatment, which preliminary indicated that TMP was a mightily competitive combination with TLR4/MD2 complex, and attributed to the activation of NF-κB, thus up-regulated HPSE1, further enhanced endothelial glycocalyx degradation and monocyte adhesion to endothelial cell.

**FIGURE 5:**
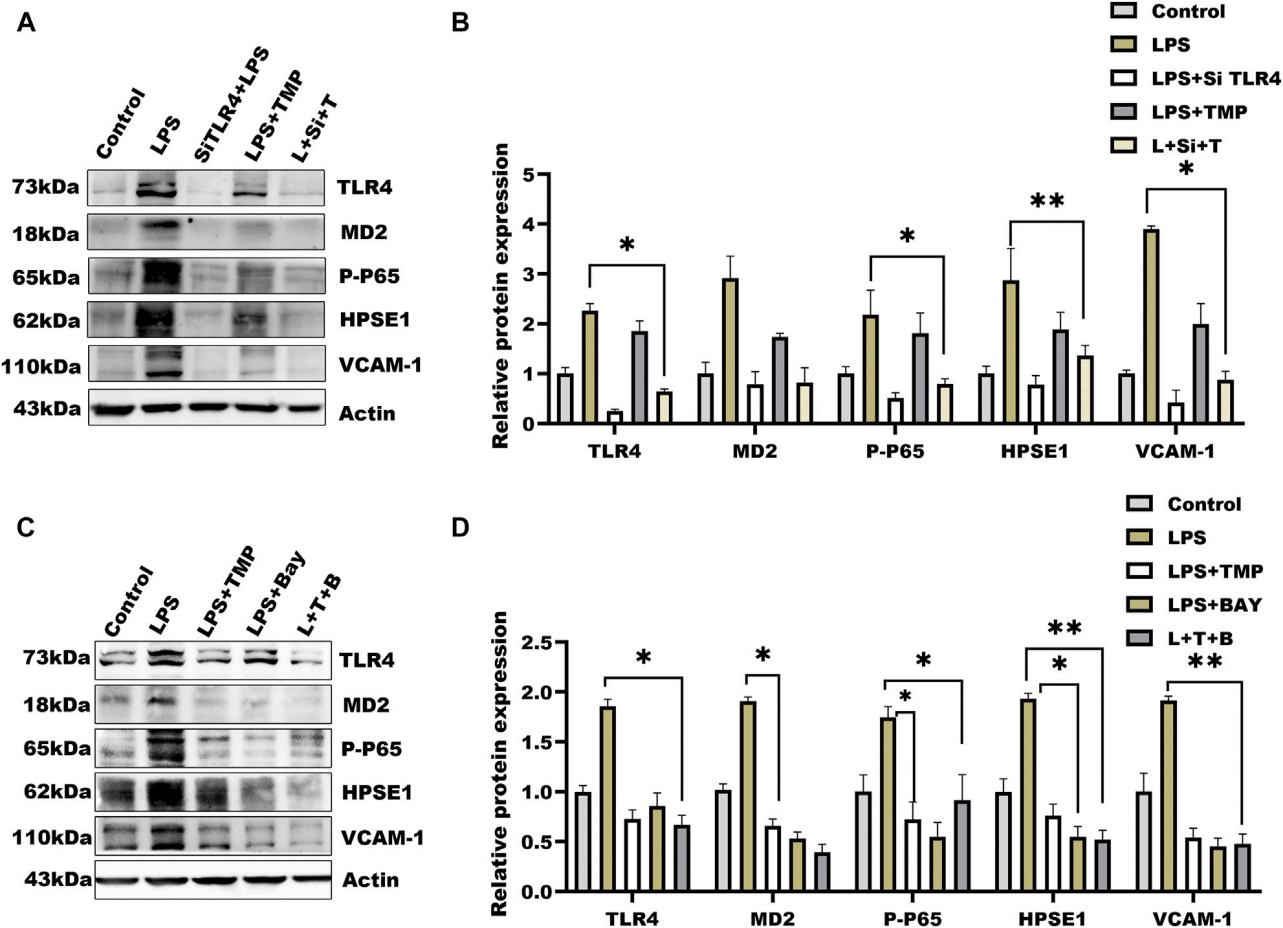
Effect of TMP combined with siRNA TLR4 or NF-κB inhibitor on expression of glycocalyx degradation associated proteins **(A)** Western blot analysis of short interference TLR4 after the HUVEC signal axis of the protein **(B)** Quantitative analysis of protein bands was performed by ImageJ software **(C)** Western blot was used to analyze the proteins on the signal axis of HUVEC after treatment with NF-κB inhibitor BAY11-7082 (20 μM) **(D)** Quantitative analysis of protein bands was performed by ImageJ software. *p < 0.05. **p < 0.005; ***p < 0.001.

Considering TMP-induced TLR4/NF-κB/HPSE1 activation, we further established cells by transferring TLR4 short interfering RNAs into cells (Supplementary Table S3). Compared with negative cells, TLR4 expression after 36 h of transfection was significantly reduced (Supplementary Figure S1E), indicating the target gene was effectively knockdown. To exclude the influence of TMP on downstream pathways, we then detected the expression of MD2, P65-NF-κB, and HPSE1. Results in Figures 5A,B showed that the above protein levels were significantly hindered, so as to the VCAM-1 levels. Similarly, the effect of TMP on TLR4/NF-κB/HPSE1 signaling was promoted in cells when using TMP united BAY11-7082, an inhibitor of NF-κB (Figures 5C,D). Interestingly, TMP almost had little influence on TLR4 signaling pathway when cells were transfected with HPSE1 over-expression plasmid (Supplementary Figure S1D). These data demonstrated that TLR4/NF-κB/HPSE1 signaling pathway played an important role in endothelial glycocalyx homeostasis upon TMP pretreatment.

### TMP Protected Endothelial Glycocalyx Degradation Against Inflammatory Stimulation *in vivo*

**FIGURE 6:**
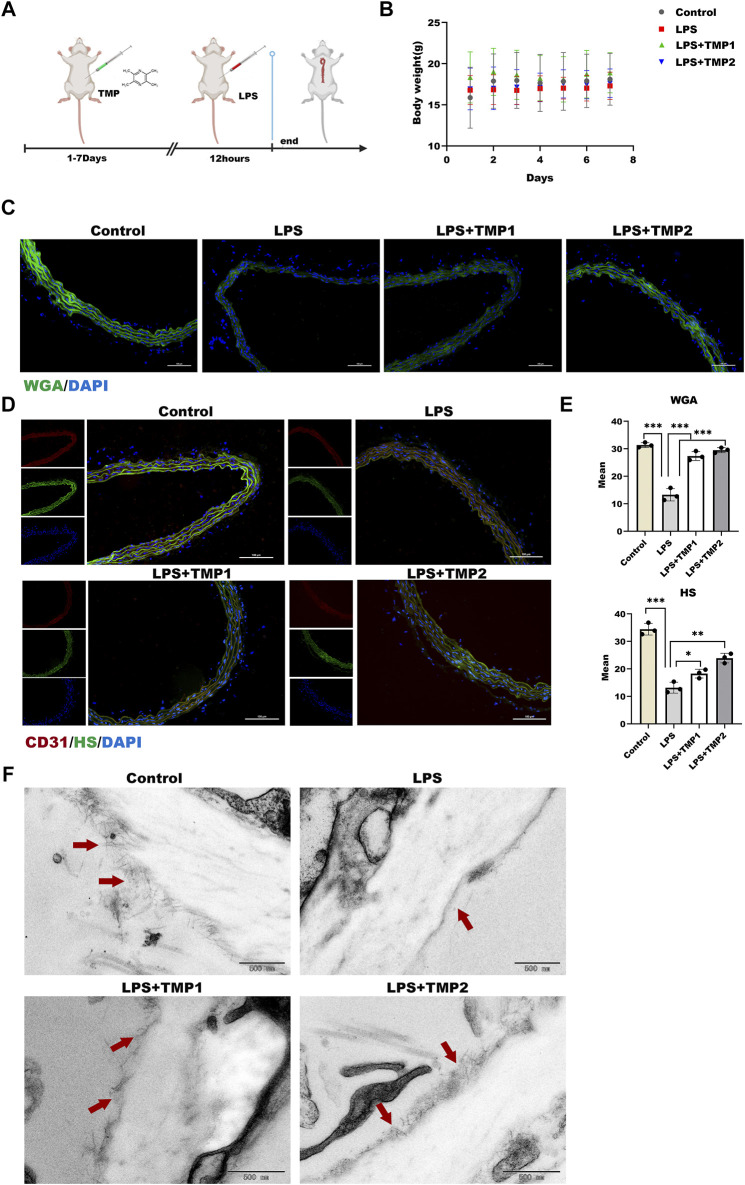
Effect of TMP on endothelial glycocalyx integrity *in vivo* **(A)** After intraperitoneal injection of TMP (3 mg/kg, 6 mg/kg) for 7 days, the mice were injected intraperitoneally with LPS (2.5 mg/kg) for 12 h. The mice were sacrifice, serum and aorta were collected **(B)** Daily body weight recording of mice during feeding period **(C)** Sections of the aorta were stained with WGA Lectin FITC (Green), DAPI (Blue) by immunofluorescence and Mean fluorescence quantitative statistic (*n* = 3) **(D)** HS (Green) expression in the aorta was expressed by immunofluorescence. CD31 (Red), DAPI (Blue) **(E)** Fluorescence of (D) and (E) was quantified by ImageJ **(F)** The glycocalyx of the aorta was photographed by transmission electron microscopy, The red arrow points to a villous glycocalyx. *p < 0.05. **p < 0.005; ***p < 0.001.

Based on the correlation between TMP and glycocalyx integrity *in vitro*, we determined the protection of TMP on endothelial glycocalyx in aortic arch tissues in an LPS exposure-induced vascular injury in mice (Figure 6A). As shown in Figure 6B, there was no significant difference in body weight and weekly food intake between the four groups throughout the experiment. Biochemical analysis manifested that the higher levels of liver injury indicators such as AST, ALT, and ALB upon LPS treatment were reduced in aortic arch tissues with TMP pretreatment to the kidney injury indicators such as BUN and CREA (Table 1). These results indicated that TMP could reverse the tissue injury during inflammation.

**TABLE 1:** Effect of LPS and TMP on the biochemical parameters.

Biochemical parameters	Control (mean ± SD)	LPS (mean ± SD)	LPS + T1 (mean ± SD)	LPS + T2 (mean ± SD)
AST (U/L)	129.97±7.19	361.97±7.80^b^	300.77±13.39^a^	282.80±13.33^a^
ALT (U/L)	56.67±13.05	147.70±7.79^b^	115.83±11.93^a^	108.22±6.12^a^
ALB (g/L)	30.43±0.51	39.62±0.97^a^	30.04±2.25	31.93±2.14^a^
BUN(mg/dl)	22.46±1.12	111.89±5.75^b^	90.24±4.78	88.59±5.85
CREA (μmol/L)	25.20±1.01	60.66±6.08^a^	37.55±2.65	33.48±7.91

All values were expressed as mean ± SD (*n* = 3).

a *p* < 0.05.

b *p* < 0.005.

c *p* < 0.001, as compared to disease control group. Results were done by two-way ANOVA, followed by Dunnett’s test.

**FIGURE 7:**
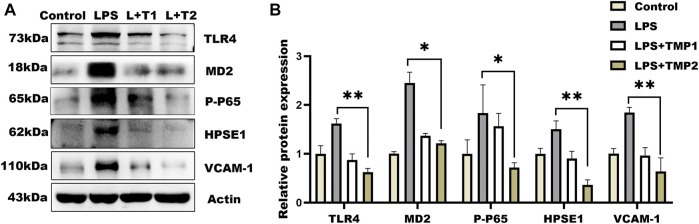
Effect of TMP on glycocalyx degradation associated proteins expression *in vivo*. After intraperitoneal injection of TMP (3 mg/kg, 6 mg/kg) for 7 days, the mice were injected intraperitoneally with LPS (2.5 mg/kg) for 12 h **(A)** Expression of TLR4 Signal Axis Associated Proteins in Mouse Aorta by Western blot Analysis **(B)** Quantitative analysis of protein bands was performed by ImageJ software. *p < 0.05. **p < 0.005; ***p < 0.001.

Moreover, glycocalyx integrity was determined by using an immunofluorescence assay. The results of WGA staining revealed higher levels of glycocalyx in vascular tissues with TMP pretreatment compared with LPS group (Figures 6C,E), as well as the HS levels shown in Figures 6D,E. Furthermore, TEM images revealed glycocalyx density and thickness in TMP-treated mouse blood vessels (Figure 7F). These results demonstrate that TMP could repair the endothelial glycocalyx degradation *in vivo.* In addition, we determined the protein levels of TLR4, NF-κB, and HPSE1. Results shown in Figures 7A,B revealed the same mechanism as *in vitro*. As the main effector of inflammatory activation, VCAM-1 expression was also inhibited upon TMP treatment. Collectively, these results provided evidence to support the clinical relevance of TMP and glycocalyx injury in inflammatory progressions.

## Discussion

The integrity of vascular endothelial glycocalyx is necessary for the maintenance of vascular endothelial function and homeostasis. Glycocalyx injury is involved in intracellular signal transduction, cytoskeleton, thrombosis, inflammation/anti-inflammatory control, and immune control (Yamaoka-Tojo, 2020). Our previous studies show that glycocalyx components decreased and aggravated the atherosclerotic process in the model of shear stress environment disorder combined with inflammatory stimulation. The present research is based on the inflammatory microenvironment in the early stage of disease, to explore how to protect the integrity of glycocalyx by TMP in atherosclerotic disease. The results confirmed that pretreatment with TMP effectively improved endothelial glycocalyx shedding through a TLR4/NF-κB/HPSE1 pathway both *in vitro* and *in vivo*, which extended our knowledge of TMP to treat diseases involving glycocalyx injury.

A lot of factors cause glycocalyx damage, such as lipopolysaccharide (LPS) (Sampei et al., 2021), hypernatremia, oxidized low-density lipoprotein (LDL), and reduced vascular wall shear stress (Yamaoka-Tojo, 2020). These stimuli systemically induce shedding of vascular endothelial glycocalyx, cause the glycocalyx layer thinning, and lead to endothelial damage. LPS acts as a pro-inflammatory substance to trigger an inflammatory response and induce monocyte into the endothelium, resulting in endothelial functional impairment (Park and Lee, 2013). LPS-induced glycocalyx damage has been a standard model of vascular injury (Liu et al., 2018; Wang et al., 2021). We evaluated the effect of LPS on glycocalyx integrity as endothelial inflammatory maker. Moreover, heparin sulfate (HS) is the main component of the endothelial glycocalyx and the context of HS on the surface of cells could be used to evaluate glycocalyx damage (Zhang et al., 2019). Vascular cell adhesion molecule 1 (VCAM-1), one of the initial and critical proteins, mediates early leukocyte attachment and rolling on the endothelial surface and is regarded as an endothelial injury maker (Li et al., 2020). Our results show that the glycocalyx structure can be significantly destroyed and trigger an inflammatory response that recruits monocytes to the endothelial surface after LPS stimulation.

Some research has shown that TMP can inhibit apoptosis and the generation of reactive oxygen species (Fan et al., 2019). Our data showed that pretreatment with TMP on HUVECs or mice followed by LPS stimulation restored glycocalyx damage and suppressed the inflammatory response. Therefore, how does TMP affect glycocalyx stability is our place emphasis on. Previous studies have shown that enzymatic degradation was involved in the endothelial glycocalyx degradation. Studies have found that inhibited heparanase activation could protect pulmonary endothelial glycocalyx integrity and reduce endothelial dysfunction (Wang et al., 2016; Huang et al., 2018; Wang et al., 2020). In the present study, the increased expression of HPSE1 together with glycocalyx injury was found in the LPS-induced model, and aggravated glycocalyx injury was induced with HPSE1 over-expression, and further enhanced inflammatory reaction and spread inflammatory factors, resulting in persistent endothelial injury. However, TMP can inhibit the expression of HPSE1, glycocalyx shedding, and neutrophil adhesion to endothelial surface.

LPS aggregates into monomeric molecules and presents them to the TLR4/MD-2 complex. Aggregation of the TLR4/MD-2 complex after binding LPS leads to activation of multiple signaling components, including NF-kB and IRF3, and the subsequent production of pro-inflammatory cytokines (Park and Lee, 2013). However, fewer reported how this response damaged the endothelial glycocalyx and accelerated the spread of inflammation. Interestingly, we found that TMP and TLR4/MD2 complex proteins can bind very tightly when we used molecular docking technology. Our present study further showed that LPS could activate TLR4/MD2/NF-κB signal and then induced the downstream response factor, such as HPSE1 down-regulation and inflammatory response, which initiate the inflammatory cascade associated with glycocalyx injury and aggravate the development of endothelial injury. Regrettably, our present study did not involve how TMP affects the HPSE1 activity, and further studies are needed to determine the mechanism of protein levels.

In the present study, the most important finding is to establish the mechanism of TMP’s protection on glycocalyx injury induced by LPS. TMP inhibits HPSE1 and weakens the degradation process in glycocalyx biosynthesis, thus playing a pharmacological role in protecting endothelial injury. These mechanisms emphasize the role of TMP in the regulation of HPSE1 and its important role in vascular injury.

## Conclusion

**FIGURE 8:**
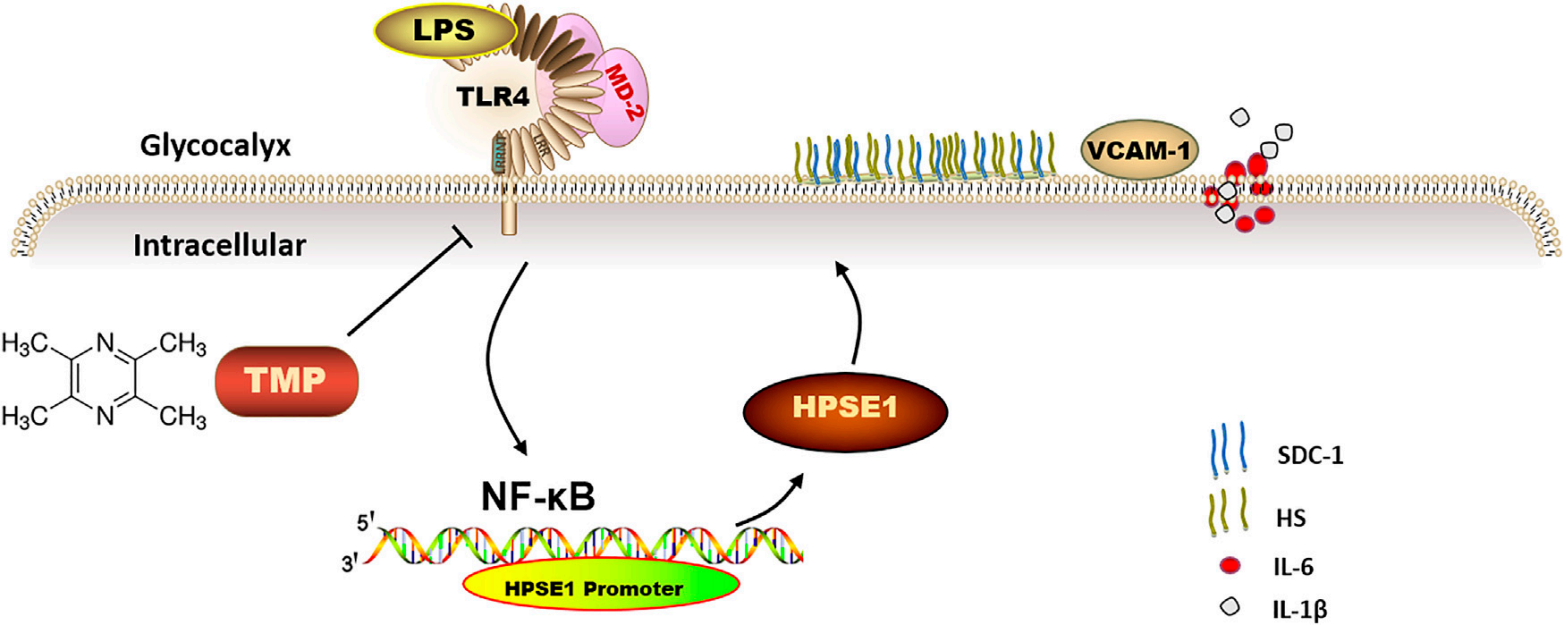
The mechanism of TMP protecting endothelial glycocalyx and maintaining endothelial function. TMP can inhibit the expression of glycocalyx degrading enzymes by inhibiting TLR4 to inhibit HPSE1 or directly inhibit HPSE1, and protect the integrity of glycocalyx structure.

This study firstly indicates that TMP reduces endothelial glycocalyx loss and improves glycocalyx reconstitution by modulating glycocalyx key enzymes HPSE1 through TLR4/MD2/NF-κB cascade after LPS treatment (Figure 8). The preservation of glycocalyx by TMP treatment can reduce the adhesion of endothelial cells, further protect endothelial function, and delay the progression of arteriosclerosis disease. These findings not only provide a potential theoretical target for TMP treatment of cardiovascular and cerebrovascular diseases but also provide therapeutic strategies for some other diseases resulting from glycocalyx damage.

## Data Availability

The original contributions presented in the study are included in the article/Supplementary Materials, further inquiries can be directed to the corresponding authors.
