# Supplementary material for: Tetramethylpyrazine Alleviates Endothelial Glycocalyx Degradation and Promotes Glycocalyx Restoration via TLR4/NF-κB/HPSE1 Signaling Pathway During Inflammation
Source: Front Pharmacol. 2022 Jan 3;12:791841. doi: 10.3389/fphar.2021.791841 (PMC8850260; doi:10.3389/fphar.2021.791841)
Supplement: Supplementary file 1 [file Table1.docx]

Supplementary Material

**Supplemental Figures**


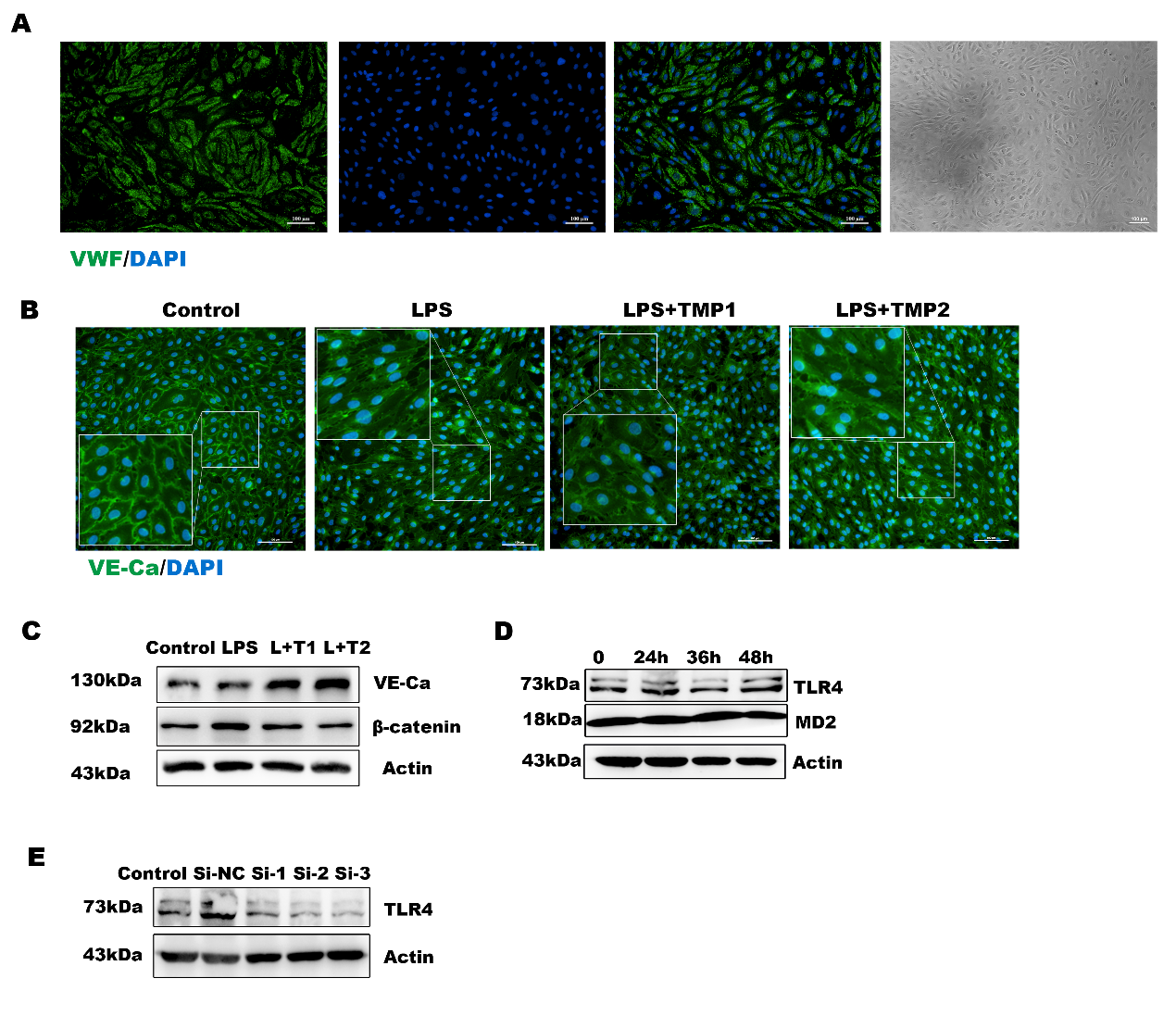


**Supplemental Figure 1.**

(A) HUVEC identification of fluorescent staining using VWF (Green), DAPI(Blue) and white light image. (B) Immunofluorescence staining of connexin between HUVEC cells using VE-Ca (Green), DAPI(Blue), (n=3). (C)Western blot analysis of the changes of connexin between cells after TMP pretreatment. (D) Western blot analysis of upstream protein expression of HUVEC after overexpression. (E) Western blot analysis of HUVEC transfection efficiency. Bar:100μm.

**Supplemental Tables**

**Supplementary Table 1 The primers used in qPCR assay.**

| Primers(homo) | Sequence (5′-3′) |
| --- | --- |
| SDC-1 | Forward GCCGCAAATTGTGGCTACT |
|  | Reverse GGTTCTGGAGACGTGGGATATG |
| HS | Forward CATGGGCTGAGGGCATACG |
|  | Reverse TGTGCCCAGGCGTCGGAAC |
| VCAM-1 | Forward CCCCAGAGATACAACCGTC |
|  | Reverse TCCACAGGATTTTCGGAGCA |
| β-Actin | Forward TTGCCGACAGGATGCAGAA |
|  | Reverse GCCGATCCACACGGAGTACT |

**Supplementary Table 2 The binding energy of Tetramethylpyrazine and TLR4-MD-2 conformation**

| **Cluster Rank** | | **Lowest Binding Energy (kcal/mol)** | **Mean Binding Energy(kcal/mol)** | **Num in Cluster** |
| --- | --- | --- | --- | --- |
| 1 | -4.75 | | -4.75 | 5 |
| 2 | -4.74 | | -4.72 | 4 |
| 3 | -4.31 | | -4.31 | 4 |
| 4 | -4.24 | | -4.24 | 2 |
| 5 | -4.12 | | -4.12 | 2 |
| 6 | -3.82 | | -3.82 | 1 |
| 7 | -3.70 | | -3.70 | 1 |
| 8 | -3.63 | | -3.63 | 1 |

**Supplementary Table 3 SiRNA primer sequences**

| Primers(homo) | Sequence (5′-3′) |
| --- | --- |
| TLR4-Si-1 | Forward GCAAUUUGACCAUUGAAGATT |
|  | Reverse UCUUCAAUGGUCAAAUUGCTT |
| TLR4-Si-2 | Forward CAUUGGAUACGUUUCCUUATT |
|  | Reverse UAAGGAAACGUAUCCAAUGTT |
| TLR4-Si-3 | Forward GAAGUUGAACGAAUGGAAUTT |
|  | Reverse AUUCCAUUCGUUCAACUUCTT |
